# Feasibility of a Deep‐Learning Model for Objective Visualization‐Quality Assessment During Cholangioscopy‐Guided Lithotripsy: A Proof‐of‐Concept Study

**DOI:** 10.1002/deo2.70454

**Published:** 2026-10-18

**Authors:** Hideki Kamada, Kiyoyuki Kobayashi, Hiroki Yamana, Naoki Fujita, Daisuke Namima, Manabu Yamada, Syunsuke Fujita, Hironobu Suto, Keiichi Okano, Hideki Kobara

**Affiliations:** ^1^ Department of Gastroenterology and Neurology, Faculty of Medicine Kagawa University Kagawa Japan; ^2^ Department of Gastroenterological Surgery, Faculty of Medicine Kagawa University Takamatsu Japan

**Keywords:** artificial intelligence, deep learning, electrohydraulic lithotripsy, gel immersion, peroral cholangioscopy

## Abstract

During peroral cholangioscopy‐guided electrohydraulic lithotripsy (EHL), stone fragmentation obscures the visual field (the “snowstorm effect”), and visualization quality has been assessed only subjectively. This proof‐of‐concept study explored the feasibility of a deep‐learning method for objectively quantifying visualization quality during EHL and, as an exploratory application, compared viscous gel immersion with saline irrigation. A ResNet‐18 model was trained on expert‐annotated frames (5‐point scale) from three development cases. For independent validation, 120 frames from 12 previously unseen comparison cases were scored by two blinded endoscopists (inter‐reader weighted *κ* = 0.98). The model showed only moderate agreement with their consensus (weighted *κ* = 0.60; accuracy 70.8%); because the validation sample was enriched for predicted‐usable frames, metrics re‐weighted to the natural frame distribution gave sensitivity 29.8% and specificity 93.5%, indicating systematic under‐detection of usable views. Agreement did not differ systematically between gel and saline frames. The High‐Quality View Rate (HQVR) was the proportion of EHL‐phase frames scored Good/Excellent. In a patient‐level comparison, median HQVR was 22.7% for gel versus 8.4% for saline (exact Mann–Whitney U, *p* = 0.15, not significant); in post hoc exploratory analyses, a threshold‐independent mean predicted score differed (1.65 vs. 1.35, *p* = 0.005) and the longest continuous usable interval favored gel (median 24.4 vs. 5.2 s). Mild cholangitis occurred in 1/5 gel and 4/7 saline patients. Training such a model is feasible, but it is not yet a validated measurement tool, and the gel‐versus‐saline signal is hypothesis‐generating only. Larger multi‐case training and prospective validation are required.

**Trial Registration**: N/A

## 1 Introduction

Single‐operator peroral cholangioscopy (POCS) has revolutionized the management of difficult bile duct stones, allowing direct visualization and precise lithotripsy [1, 2]. Electrohydraulic lithotripsy (EHL) is widely used under POCS guidance; however, maintaining a clear visual field remains a significant technical hurdle. The shockwaves generated during EHL rapidly fragment stones into fine debris and sludge that, combined with air bubbles and bile, instantly obscure the endoscopic lens — analogous to the “snowstorm effect” described in urological laser lithotripsy [3, 4].

This deterioration often forces the endoscopist to interrupt the procedure for vigorous saline irrigation, prolonging the procedure; excessive irrigation can also raise intrabiliary pressure and increase the risk of cholangitis or bacteremia [5]. In severe cases, lithotripsy may proceed under suboptimal visualization, increasing the risk of bile duct wall injury.

Saline, the conventional immersion and irrigation medium, has low viscosity that allows turbulent dispersion of debris during shockwave delivery. A transparent, high‐viscosity gel, originally introduced to secure the visual field in acute gastrointestinal bleeding, has since shown promise in several endoscopic applications [6, 7]; because it resists rapid mixing with fluids [6], it may suppress the turbulent dispersion of debris during EHL.

A major obstacle to testing this hypothesis is the absence of objective measures of visualization quality, which has relied on subjective Likert scales; deep learning offers a route to objective image analysis [8, 9]. The primary aim of this proof‐of‐concept study was to explore the feasibility of a deep‐learning model that objectively quantifies visualization quality during EHL, and to characterize its performance honestly, including independent validation against expert labels. As a secondary, exploratory application, we used the model to compare viscous gel immersion with saline.

## 2 Procedure

### 2.1 Study Design and Patient Selection

This retrospective pilot analysis at Kagawa University reviewed records and video archives of patients who underwent POCS‐guided EHL for difficult bile duct stones between November 2021 and June 2025. In total, 15 patients were included (patient flow, Figure S3): 3 development cases, used only for model training and a convergence check, and 12 comparison cases forming the test set, with no overlap. The development cases were the first three consecutive procedures in which both viscous gel and saline were used within the same procedure, selected before the comparison cohort was assembled and solely on this mixed‐medium criterion (Table S1). The 12 comparison cases were categorized by the medium used — Viscous Gel (*n* = 5) or Saline (*n* = 7) — and each was irrigated with a single medium throughout the analyzed EHL phase. Patients with missing video were excluded. The protocol was approved by the Institutional Review Board (No. 2025–238) and conducted per the Declaration of Helsinki; written or opt‐out consent was obtained, including explanation of the off‐label use of the gel in the bile duct.

### 2.2 Endoscopic Procedures

All procedures used the same digital single‐operator cholangioscope (SpyGlass DS II; Boston Scientific, Marlborough, MA, USA) and the Autolith Touch EHL system (Northgate Technologies, Elgin, IL, USA; generator M00546800; 1.9‐Fr, 375‐cm probe M00546620). In the Saline group, the duct was irrigated with normal saline; in the Viscous Gel group, a dedicated endoscopic gel (VISCOCLEAR; Otsuka Pharmaceutical Factory, Tokushima, Japan) was injected manually via the accessory channel before and during lithotripsy as needed. Owing to the retrospective design, generator settings, irrigation flow/pressure, and injected gel volume were not recorded, and injection and irrigation were not protocolized across operators (details in Supporting Information Methods).

### 2.3 Dataset Preparation and Artificial Intelligence Model Development

Training frames were extracted from the 3 development cases; because all three contained both gel and saline segments, the model was trained on both media. Frames were labeled by score only, and because the media alternated within each procedure, individual training frames cannot be reliably attributed to a medium; the empirical check that the model does not score one medium differently is provided by the per‐medium independent validation (Section 3.2, Table 3). A 100‐frame hold‐out set sampled from the same development cases served only as a convergence check: because near‐duplicate neighboring frames remained in training, it cannot demonstrate generalization and may overestimate performance. The test set comprised the EHL‐phase clips of the 12 comparison patients, unseen during training.

A 5‐point scale was used (Score 0, Unusable; 1, Poor; 2, Fair; 3, Good; 4, Excellent; Figure S1). One board‐certified POCS expert (Reader A) assigned all training labels; two additional endoscopists blindly scored a disjoint subset to assess the scale (weighted *κ* 0.60–0.79), which validates the scale rather than the training labels. The *κ* subset, hold‐out set, and accuracy‐evaluation set were mutually disjoint, with Reader A's annotations as the reference throughout.

Training configuration. ResNet‐18 [10] initialized with ImageNet [11]weights (torchvision IMAGENET1K_V1) with a 5‐unit classifier was trained with cross‐entropy loss and the Adam optimizer (learning rate 1×10^−^^4^, weight decay 1×10^−^^4^; batch size 8; 20 epochs; best‐validation checkpoint). Class imbalance (Score 0/1/2/3/4 = 45/964/465/202/56 frames) was addressed with an inverse‐frequency weighted random sampler, not augmentation. Input pre‐processing, augmentation, and the 80/20 split are given in the Supporting Information Methods. Reporting follows applicable CLAIM and TRIPOD+AI items [12, 13]; unmet items are listed in the Limitations.

#### 2.3.1 Independent Validation on Unseen Comparison Cases

To test generalizability without retraining, 120 frames (10 per case) from the 12 comparison cases were scored by two board‐certified POCS endoscopists blinded to group, medium, and model output; the reference was their per‐frame consensus. Frames were drawn at random while cycling through the model's predicted classes so that each class was represented, which enriched the sample for predicted‐usable frames (39% versus 13.8% of all 1950 analyzed frames). Because selection depended on model output, raw accuracy, sensitivity, specificity, and *κ* from this sample are subject to verification bias; per‐predicted‐class positive predictive values are unbiased, so we also report binary metrics re‐weighted to the natural predicted‐class distribution (Begg–Greenes‐type correction; bootstrap 95% confidence intervals [CIs]) and results by medium (Supporting Information Methods). Metrics comprised weighted *κ* (5‐class), binary accuracy/sensitivity/specificity (Usable 3–4 vs. Unusable 0–2) with Wilson 95% CIs, and per‐class precision, recall, and F1 (Table 2).

### 2.4 Quantitative Video Analysis

The model then analyzed each comparison case's EHL‐phase clip, defined as the EHL duration — from initial probe activation to final fragmentation of the target stone, measured from the video (Table 1) — including brief intra‐phase pauses. From each clip, 150–220 frames were sampled at equal intervals (1.1–10.9 s depending on clip length; 1950 frames in total), replacing the 0.2‐s sampling of the original analysis to reduce temporal autocorrelation. For each frame, the model produced class probabilities. High‐Quality View Rate (HQVR) was defined per patient as the proportion of sampled frames whose combined probability for Score 3+4 exceeded 50%; no validated clinical threshold exists, so HQVR is interpreted as a relative between‐media measure. As post hoc exploratory measures, we computed a threshold‐independent mean predicted quality score (MPQS; probability‐weighted expected score, 0–4) and temporal measures (time to first sustained snowstorm and longest continuous usable interval; resolution equal to the sampling interval). Group comparisons used the exact Mann–Whitney U test at the patient level (*n* = 5 vs. 7), reported as medians with interquartile ranges; MPQS and temporal analyses were not pre‐specified.

**TABLE 1:** Baseline characteristics of the patients and stones.

Characteristic	Viscous gel (*n* = 5)	Saline (*n* = 7)
Age, years, mean ± SD	73.0 ± 1.4	71.1 ± 18.6
Sex (M/F)	3/2	4/3
Stone size, mm, mean ± SD	16.2 ± 5.6	13.3 ± 5.7
Multiple stones, *n* (%)	5 (100%)	5 (71.4%)
EHL duration, s, median (range)	928 (532–1563)	455 (248–1660)
Mild cholangitis (TG18 Grade I), *n* (%)	1 (20.0%)	4 (57.1%)

No formal statistical testing was performed for baseline characteristics owing to the small sample and varying stone burden; non‐significance must not be read as group comparability. EHL duration is defined as the interval from initial probe activation to final fragmentation of the target stone, measured from the procedure video; it is identical to the analyzed video clip (Section 2.4). SD, standard deviation; EHL, electrohydraulic lithotripsy; TG18, Tokyo Guidelines 2018.

## 3 Results

### 3.1 Baseline Characteristics

Baseline characteristics are summarized in Table 1. Formal hypothesis testing of baseline variables was not performed given the small sample; accordingly, the absence of a significant difference should not be interpreted as evidence that the groups were comparable. Notably, the gel group had larger stones (16.2 vs. 13.3 mm) and longer EHL duration (median 928 vs. 455 s), suggesting more challenging cases — a confounder that biases against, rather than toward, the efficiency rationale for gel. Mild cholangitis (Tokyo Guidelines 2018, Grade I) occurred in 4/7 (57.1%) saline versus 1/5 (20.0%) gel patients; all resolved with conservative treatment, with no severe procedure‐related adverse events.

### 3.2 Model Performance

Primary (independent validation on unseen cases). The two blinded readers agreed almost perfectly (5‐class weighted *κ* = 0.98). Against their consensus, the model showed moderate agreement (weighted *κ* = 0.60). On the enriched sample, binary accuracy was 70.8% (Wilson 95% CI 62.2–78.2), sensitivity 60.7% (34/56; 47.6–72.4), and specificity 79.7% (51/64; 68.3–87.7) (Table 2 and Figure 1). Re‐weighted to the natural distribution of all 1950 analyzed frames, estimated accuracy was 73.6% (bootstrap 95% CI 63.2–83.0), sensitivity 29.8% (21.2–43.2), and specificity 93.5% (90.4–96.3), with an estimated true‐usable prevalence of 31.3% versus 13.8% predicted: under real‐world conditions the model is specific but markedly under‐sensitive, so HQVR underestimates usable time in both groups. Per medium (Table 3), agreement was moderate for both gel (*n* = 50; *κ* = 0.45; accuracy 72.0%) and saline frames (*n* = 70; *κ* = 0.69; accuracy 70.0%), with overlapping CIs; the mean signed error was −0.40 for gel and −0.19 for saline (permutation *p* = 0.34), i.e., no significant medium‐specific bias, and if anything the model scored gel more conservatively, which biases against the observed gel advantage. These independent metrics fall well below the hold‐out values, confirming that the hold‐out overestimated real‐world performance.

**TABLE 2:** Independent validation — Model versus blinded expert consensus (120 frames and 12 unseen cases).

Predicted score	Precision	Recall	F1	Support (*n*)
Score 0 (Unusable)	0.59	0.56	0.57	18
Score 1 (Poor)	0.25	0.47	0.33	17
Score 2 (Fair)	0.29	0.24	0.26	29
Score 3 (Good)	0.36	0.39	0.38	31
Score 4 (Excellent)	0.43	0.24	0.31	25

Binary Usable (3–4) vs. Unusable (0–2), model vs. consensus: accuracy 70.8% (Wilson 62.2–78.2), sensitivity 60.7% (34/56; 47.6–72.4), specificity 79.7% (51/64; 68.3–87.7). Five‐class weighted *κ*: reader1–reader2 = 0.98, model–consensus = 0.60. Binary (0–2 vs. 3–4) Cohen *κ*: reader1–reader2 = 0.95, model–consensus = 0.41. Re‐weighted to the natural predicted‐class distribution of all 1,950 analyzed frames (verification‐bias correction): accuracy 73.6% (bootstrap 95% CI 63.2–83.0), sensitivity 29.8% (21.2–43.2), specificity 93.5% (90.4–96.3); estimated prevalence of truly usable frames 31.3%.

**FIGURE 1:**
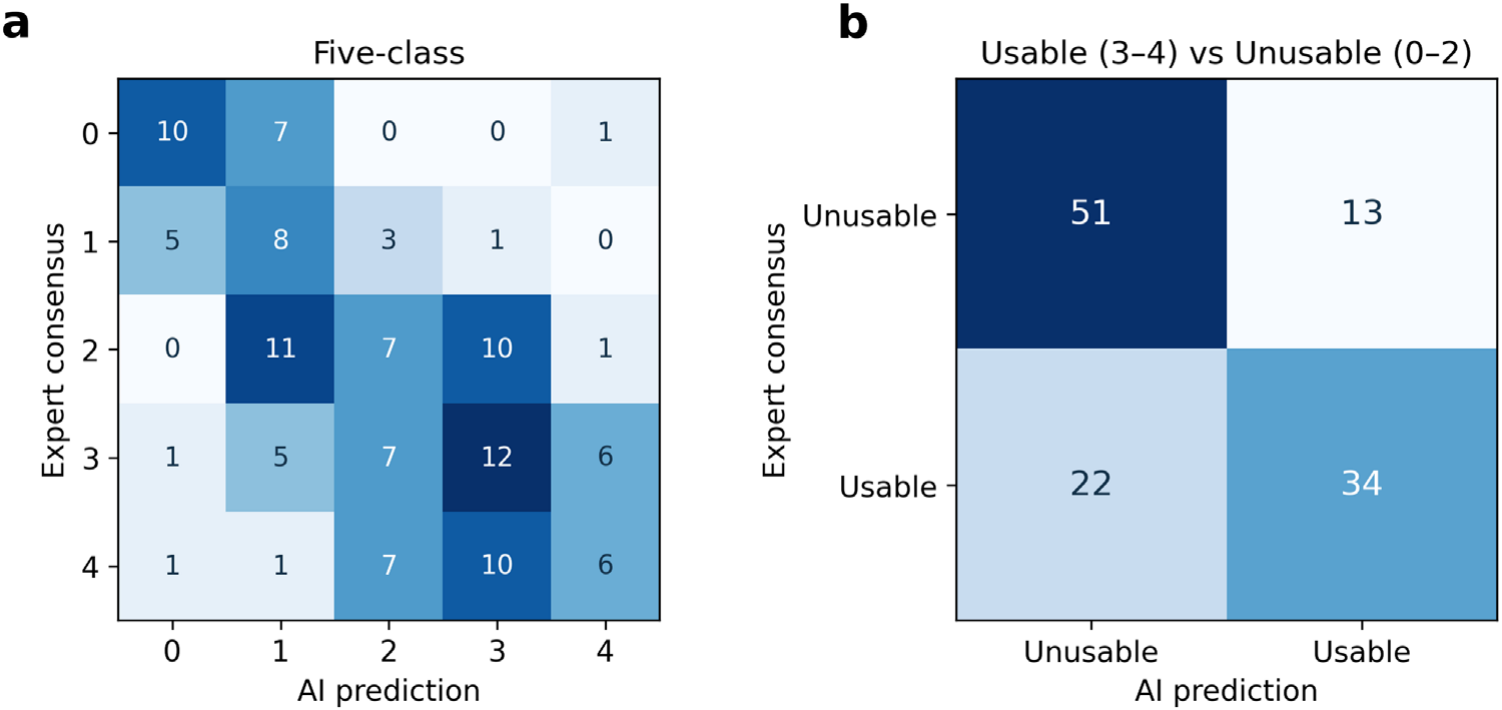
Independent validation of the deep‐learning model on 120 randomly sampled frames from the 12 previously unseen comparison cases, scored by two blinded endoscopists. (a) Five‐class confusion matrix and (b) binary (Usable, Score 3–4 vs. Unusable, Score 0–2) confusion matrix, model versus expert consensus. Overall accuracy 70.8%, sensitivity 60.7%, specificity 79.7%; five‐class weighted *κ* = 0.60, binary *κ* = 0.41. Inter‐reader agreement was near‐perfect (weighted *κ* = 0.98).

**TABLE 3:** Independent validation by immersion medium (model vs. blinded expert consensus).

Metric	Gel frames (*n* = 50)	Saline frames (*n* = 70)
Inter‐reader weighted *κ*	0.98	0.98
Model–consensus weighted *κ* (5‐class)	0.45	0.69
Accuracy, % (Wilson 95% CI)	72.0 (58.3–82.5)	70.0 (58.5–79.5)
Sensitivity, % (*n*)	62.1 (18/29)	59.3 (16/27)
Specificity, % (*n*)	85.7 (18/21)	76.7 (33/43)
Mean signed error, model − consensus (SD)	−0.40 (1.29)	−0.19 (1.03)
Predicted‐usable rate/consensus‐usable rate	42%/58%	37%/39%

Difference in mean signed error between media: permutation test *p* = 0.34. Sensitivity/specificity are computed on the prediction‐stratified sample and are subject to the same verification bias as Table 2.

Convergence check. On the 100‐frame hold‐out set, binary accuracy was 99.0%, sensitivity 100% (16/16; Wilson 79.4%–100%), and specificity 98.8% (83/84), whereas the best validation accuracy during training was 79.5%; the higher hold‐out figures arise because those frames overlap the training videos and do not establish generalizability.

### 3.3 Comparative Analysis of Visualization Quality

In this exploratory, patient‐level comparison (Figure 2c), median HQVR was 22.7% (interquartile range 19.3–24.0) for gel versus 8.4% (6.7–10.1) for saline, not significant at the patient level (exact Mann–Whitney U = 27, *p* = 0.15). In post hoc exploratory analyses, MPQS differed (gel 1.65 [1.61–1.66] vs. saline 1.35 [1.23–1.37]; U = 34, *p* = 0.005; Figure 2d), suggesting the gel signal is not merely an artifact of the 50% cutoff, although with n = 12 this *p* value should not be read as confirmatory; the longest continuous usable interval was also longer with gel (median 24.4 s [20.6–26.8] vs. 5.2 s [4.4–14.4]; Figure S2). Saline visualization typically fell to Score 0–1 immediately upon lithotripsy, whereas gel sustained usable views for longer continuous periods.

**FIGURE 2:**
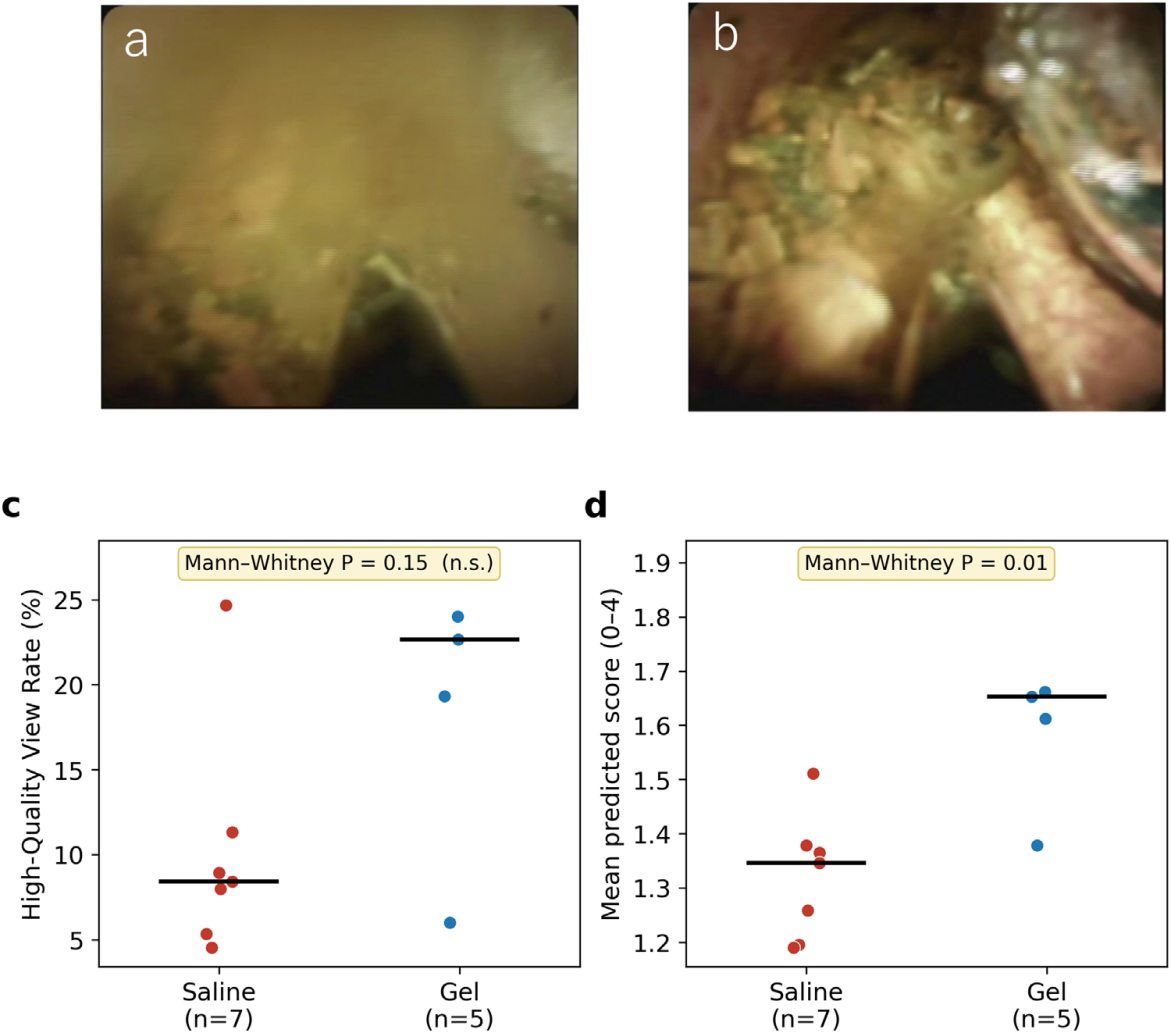
Comparative assessment of visualization quality during electrohydraulic lithotripsy (EHL). (a) Saline group: a representative frame demonstrating the “snowstorm effect.” (b) Viscous gel group: a representative frame demonstrating the stabilizing effect of the gel. (c, d) Patient‐level comparison between gel and saline (*n* = 5 vs. 7): (c) High‐Quality View Rate and (d) mean predicted quality score. Horizontal bars, group medians; *p*‐values, exact two‐sided Mann–Whitney U test. High‐Quality View Rate (HQVR) did not differ significantly (*p* = 0.15), whereas the threshold‐independent mean predicted score did (*p* = 0.005).

## 4 Discussion

In this proof‐of‐concept study, a deep‐learning model provided an objective measure of intraprocedural visualization quality, but independent validation on unseen cases showed only moderate agreement with experts (weighted *κ* = 0.60), far below the within‐training‐case hold‐out, and marked under‐sensitivity at the natural frame distribution. The model should therefore be regarded as a promising but not‐yet‐validated instrument requiring a larger, multi‐case training set, and the gel‐versus‐saline comparison — made with this imperfect instrument applied equally to both groups — remains exploratory and relative.

Mechanistically, the viscous gel resists rapid mixing with fluids [6]; in the narrow biliary lumen, low‐viscosity saline is prone to EHL‐induced turbulence that disperses debris (the snowstorm effect), whereas the gel likely maintains near‐laminar flow or acts as a physical damper, keeping the lens vicinity clear.

Clinically, a higher HQVR could plausibly contribute to safer, more efficient lithotripsy, but this study was not designed to test that, and our data do not show that higher HQVR translates into greater efficiency, less irrigation, or fewer adverse events; moreover, the gel group comprised harder cases (larger stones, longer duration), confounding any efficiency interpretation. The gel may also be useful for observational cholangioscopy, as it prevents mixing of blood and bile [6, 7].

Our study has limitations. First, it is a single‐center pilot with a very small sample (*n* = 12), precluding adjustment for confounders such as stone hardness; the gel group also had larger stones and longer procedures. Second, a temporal bias exists: saline cases are mainly earlier and gel cases more recent, allowing operator learning‐curve effects. Third, the development set comprised only three cases, limiting the diversity of learned appearances, although each contained both media and the per‐medium validation showed no systematic bias. Fourth, the hold‐out cannot show generalization and overestimated performance. Fifth, validation frames were selected by stratifying on model predictions, so raw metrics are subject to verification bias; re‐weighted estimates show the model is specific but under‐sensitive (≈30%), so HQVR underestimates usable time in both groups. Sixth, the MPQS and temporal analyses were post hoc in 12 patients, and their *p*‐values are exploratory. Seventh, frames are temporally autocorrelated, so all comparisons were made at the patient level. Eighth, HQVR has no validated clinical threshold and is not linked to outcomes. Ninth, training labels rest on a single expert reader. Several CLAIM/TRIPOD+AI items (sample‐size justification, calibration, a large independent development cohort) remain unmet.

In conclusion, training a deep‐learning model to quantify visualization quality during EHL is feasible, but independent validation showed only moderate agreement with blinded expert labels, so the model is an early feasibility demonstration rather than a validated measurement tool. Its exploratory application produced a signal consistent with gel maintaining a clearer field than saline, but this comparison is hypothesis‐generating, was not statistically robust at the patient level, and may be confounded. Larger, multi‐case development with prospective, outcome‐linked validation is required.

## Author Contributions

**Hideki Kamada**: conceptualization, methodology, investigation, writing – original draft, and project administration. **Hiroki Yamana**, **Kiyoyuki Kobayashi**, **Naoki Fujita**, and **Daisuke Namima**: investigation, data curation, and resources. **Manabu Yamada**, **Syunsuke Fujita**, **Hironobu Suto**, and **Keiichi Okano**: investigation, data curation, and validation. **Hideki Kobara**: supervision, writing – review, and editing. All the authors approved the final version of the manuscript.

## Funding

The authors have nothing to report.

## Ethics Statement

**Approval of the Research Protocol by an Institutional Review Board**: This study was approved by our institution's ethics committee (approval no. 2025–238).

## Consent

Informed consent was obtained from all patients or their legal representatives. Given the retrospective nature of the study and the absence of identifiable patient information, study details were made available on the hospital website, allowing patients to opt out if desired.

## Conflicts of Interest

The authors declare no conflicts of interest.

## Supporting information




**FIGURE S1** Definition of the 5‐point visualization scoring system, with representative endoscopic images for each score.

**FIGURE S2** Per‐case time‐course of the AI‐predicted expected score (0–4) across the analyzed EHL‐phase clip. The x‐axis is time in seconds from initial probe activation; each trace spans the EHL duration of that case as defined in Table 1 (initial activation to final fragmentation, up to ≈1,660 s), including intra‐phase pauses. Points are the sampled frames (interval 1.1–10.9 s per clip); dashed line, usable threshold (2.5).

**FIGURE S3** Patient flow. Fifteen patients underwent POCS‐guided EHL in the study period with available video; 3 mixed‐medium procedures formed the development set (training and convergence check), and the remaining 12 single‐medium procedures formed the comparison set (viscous gel, n = 5; saline, n = 7). There was no overlap between the sets.

**SUPPORTING INFORMATION METHODS** Detailed endoscopic procedure and device settings; image pre‐processing, data augmentation, and train/validation split; selection procedure for the 120 validation frames; verification‐bias correction (re‐weighting to the natural predicted‐class distribution with bootstrap confidence intervals); and per‐medium validation statistics.
