# Supplementary material for: Feasibility of a Deep‐Learning Model for Objective Visualization‐Quality Assessment During Cholangioscopy‐Guided Lithotripsy: A Proof‐of‐Concept Study
Source: DEN Open. 2026 Oct 18;7(1):e70454. doi: 10.1002/deo2.70454 (PMC13678035; doi:10.1002/deo2.70454)
Supplement: Supplementary file 1 [file DEO2-7-e70454-s001.docx]

**Supporting Information**

**Supplementary Methods**

Feasibility of a Deep-Learning Model for Objective Visualization-Quality Assessment during Cholangioscopy-Guided Lithotripsy: A Proof-of-Concept Study

**S1. Endoscopic procedure and devices**

All procedures used the same digital single-operator cholangioscope (SpyGlass DS II; Boston Scientific, Marlborough, MA, USA) inserted through a therapeutic duodenoscope. EHL was performed with the Autolith Touch EHL system (Northgate Technologies, Elgin, IL, USA; distributed by Boston Scientific), comprising the Autolith Touch EHL generator (M00546800) and a 1.9-Fr, 375-cm EHL probe for SpyGlass (M00546620). Per the manufacturer’s guidance, the probe tip was positioned approximately 1–2 mm from the stone surface and shockwaves were delivered in short bursts (approximately 3–5 pulses per activation), with output increased only as needed. Exact per-case generator settings were not recorded owing to the retrospective design. In the Saline group, the bile duct was irrigated with normal saline; the exact irrigation flow rate and pressure were not recorded. In the Viscous Gel group, a dedicated endoscopic gel (VISCOCLEAR; Otsuka Pharmaceutical Factory, Tokushima, Japan) was injected manually via the accessory channel to fill the peri-stone lumen before and during lithotripsy as needed; injected volume and timing were not recorded. Injection and irrigation were not fully protocolized across operators.

**S2. Image pre-processing, augmentation, and data split**

Images were resized and normalized with ImageNet statistics (mean [0.485, 0.456, 0.406], SD [0.229, 0.224, 0.225]). Training augmentation comprised Resize(256) followed by RandomResizedCrop(224, scale 0.8–1.0), random horizontal and vertical flips, random rotation (±30°), and color jitter (brightness/contrast/saturation 0.3); the validation transform was a plain resize to 224×224 with the same normalization. The development frames were split 80/20 at random into training and validation subsets; the checkpoint with the best validation accuracy (79.5%, 5-class) was retained. Class imbalance was addressed with a weighted random sampler using inverse class-frequency weights rather than by augmentation. No fixed random seed was set.

**S3. Selection of the 120 independent-validation frames**

From each of the 12 comparison cases, 10 frames were selected from the sampled frames (150–220 per case) by cycling through the model’s predicted classes in the order 3, 1, 2, 0, 4 and drawing one frame at random from each class in turn until 10 were obtained; classes absent in a case were skipped. This yielded 120 frames with predicted-class counts of 17/32/24/33/14 (Scores 0–4), i.e., 39% predicted usable, compared with 13.8% of all 1,950 analyzed frames (natural distribution 2.5%/68.7%/15.1%/13.1%/0.7%). Frames were anonymized and shuffled before scoring. Two board-certified POCS endoscopists, blinded to case, medium, and model output, independently scored each frame on the 5-point scale; the reference standard was the per-frame consensus (mean of the two scores, rounded).

**S4. Verification-bias correction**

Because frame selection depended on the model’s predicted class, the raw accuracy, sensitivity, specificity, and κ computed on the validation sample are not unbiased estimates for the natural frame distribution (verification bias). Under prediction-stratified sampling, the probability that a frame is truly usable given its predicted class, P(usable | predicted class c), is estimated without bias within each class. We therefore re-weighted the binary confusion matrix to the natural predicted-class distribution of all 1,950 analyzed frames (a Begg–Greenes-type correction): P(usable) = Σc P(pred = c)·P(usable | pred = c); sensitivity = Σc≥3 P(pred = c)·P(usable | pred = c) / P(usable); specificity = Σc≤2 P(pred = c)·[1 − P(usable | pred = c)] / [1 − P(usable)]. Estimated P(usable | pred = c) for c = 0–4 was 0.12, 0.19, 0.58, 0.67, and 0.86. Confidence intervals were obtained by bootstrap resampling of validation frames within each predicted class (4,000 resamples). The corrected estimates were accuracy 73.6% (95% CI 63.2–83.0), sensitivity 29.8% (21.2–43.2), and specificity 93.5% (90.4–96.3), with an estimated prevalence of truly usable frames of 31.3%.

**S5. Per-medium validation statistics**

Validation metrics were computed separately for gel (n = 50) and saline (n = 70) frames (Table 3). The difference between media in the mean signed error (model score minus consensus score) was tested with a two-sided permutation test (20,000 label permutations): gel −0.40 (SD 1.29) versus saline −0.19 (SD 1.03), difference −0.21, P = 0.34.

**S6. Frame sampling for the comparative analysis**

Each comparison case’s clip spanned its EHL duration (initial probe activation to final fragmentation, measured from the video; Table 1). Frames were sampled at equal intervals across the full clip: 150 frames for clips longer than 450 s and 190–220 frames for shorter clips, giving sampling intervals of 1.1–10.9 s (s1 10.9; s3 4.0; s4 1.1; s5 1.3; s6 1.3; s7 3.0; s8 4.9; g1 10.3; g2 3.5; g3 6.1; g4 5.4; g5 8.6 s) and 1,950 frames in total. The temporal resolution of the time-based measures equals the per-clip interval.
